# Modular systems metabolic engineering enables balancing of relevant pathways for l-histidine production with *Corynebacterium glutamicum*

**DOI:** 10.1186/s13068-019-1410-2

**Published:** 2019-03-25

**Authors:** Andreas Schwentner, André Feith, Eugenia Münch, Judith Stiefelmaier, Ira Lauer, Lorenzo Favilli, Christoph Massner, Johannes Öhrlein, Bastian Grund, Andrea Hüser, Ralf Takors, Bastian Blombach

**Affiliations:** 1 Institute of Biochemical Engineering, University of Stuttgart, Allmandring 31, 70569 Stuttgart, Germany; 2 Evonik Creavis GmbH, Paul-Baumann-Straße 1, 45772 Marl, Germany; 3 Evonik Nutrition & Care GmbH, Kantstraße 2, 33790 Halle, Germany; 4 Microbial Biotechnology, Campus Straubing for Biotechnology and Sustainability, Technical University of Munich, Straubing, Germany

**Keywords:** Modularized metabolic engineering, LC/MS-QToF-based systems metabolic profiling (SMP), Flux balance analysis (FBA), Energy engineering, l-Histidine production, *Corynebacterium glutamicum*

## Abstract

**Background:**

l-Histidine biosynthesis is embedded in an intertwined metabolic network which renders microbial overproduction of this amino acid challenging. This is reflected in the few available examples of histidine producers in literature. Since knowledge about the metabolic interplay is limited, we systematically perturbed the metabolism of *Corynebacterium glutamicum* to gain a holistic understanding in the metabolic limitations for l-histidine production. We, therefore, constructed *C. glutamicum* strains in a modularized metabolic engineering approach and analyzed them with LC/MS-QToF-based systems metabolic profiling (SMP) supported by flux balance analysis (FBA).

**Results:**

The engineered strains produced l-histidine, equimolar amounts of glycine, and possessed heavily decreased intracellular adenylate concentrations, despite a stable adenylate energy charge. FBA identified regeneration of ATP from 5-aminoimidazole-4-carboxamide ribonucleotide (AICAR) as crucial step for l-histidine production and SMP identified strong intracellular accumulation of inosine monophosphate (IMP) in the engineered strains. Energy engineering readjusted the intracellular IMP and ATP levels to wild-type niveau and reinforced the intrinsic low ATP regeneration capacity to maintain a balanced energy state of the cell. SMP further indicated limitations in the C_1_ supply which was overcome by expression of the glycine cleavage system from *C. jeikeium*. Finally, we rerouted the carbon flux towards the oxidative pentose phosphate pathway thereby further increasing product yield to 0.093 ± 0.003 mol l-histidine per mol glucose.

**Conclusion:**

By applying the modularized metabolic engineering approach combined with SMP and FBA, we identified an intrinsically low ATP regeneration capacity, which prevents to maintain a balanced energy state of the cell in an l-histidine overproduction scenario and an insufficient supply of C_1_ units. To overcome these limitations, we provide a metabolic engineering strategy which constitutes a general approach to improve the production of ATP and/or C_1_ intensive products.

## Background

l-Histidine (further referred to as histidine) was discovered in the late nineteenth century by Kossel and Hedin simultaneously [89] and the l-enantiomer is nowadays categorized as an essential amino acid for human infants and adults, belonging to the 20 standard proteinogenic amino acids [52]. Histidine has the ability to switch between the protonated and unprotonated states due to the p*K*_a_ of about 6 of its imidazole group [64]. Thus, histidine is a common ligand of metalloproteins and part of the catalytic triad in several enzymes, underlining its physiologically prominent role [57, 70, 72]. Exceeding physiological levels of histidine in humans has shown to be connected with mutations in histidase and was named histidinemia, a benign inborn error of metabolism [6, 49]. Furthermore, histidine is a precursor for histamine, which is known to play an important role in regulating human immune response, and thus is linked to several allergic disorders [67, 69]. Beyond this, histidine is available as feed supplement and has been reported to have anti-inflammatory and antioxidant properties, which makes it attractive for applications in the medical industry [25, 33, 34, 87, 90, 91].

*Corynebacterium glutamicum* is a Gram-positive, facultatively anaerobic bacterium which can grow on a wide range of sugars, alcohols, and organic acids [58, 65] and is known as a workhorse for the production of l-glutamate and l-lysine [8, 23, 83]. Moreover, metabolic engineering approaches expanded the product portfolio to other amino acids such as l-methionine, l-valine, l-arginine, and l-tryptophan [8, 42, 66, 68, 71], organic acids [17, 53, 94, 95], alcohols [13, 43, 46, 80], vitamins [40], carotenoids [35, 36], fatty acids [82], polymers [59], terpenes [26, 48], and others. Most relevant, *C. glutamicum* possesses an intrinsic histidine synthesis pathway but, in contrast to other industrially relevant bacteria such as *Pseudomonas* and several *Bacillus* genera, lacks a histidine utilization system (reviewed in [11]). This renders *C.* *glutamicum* as an attractive platform for histidine production.

**Fig. 1:**
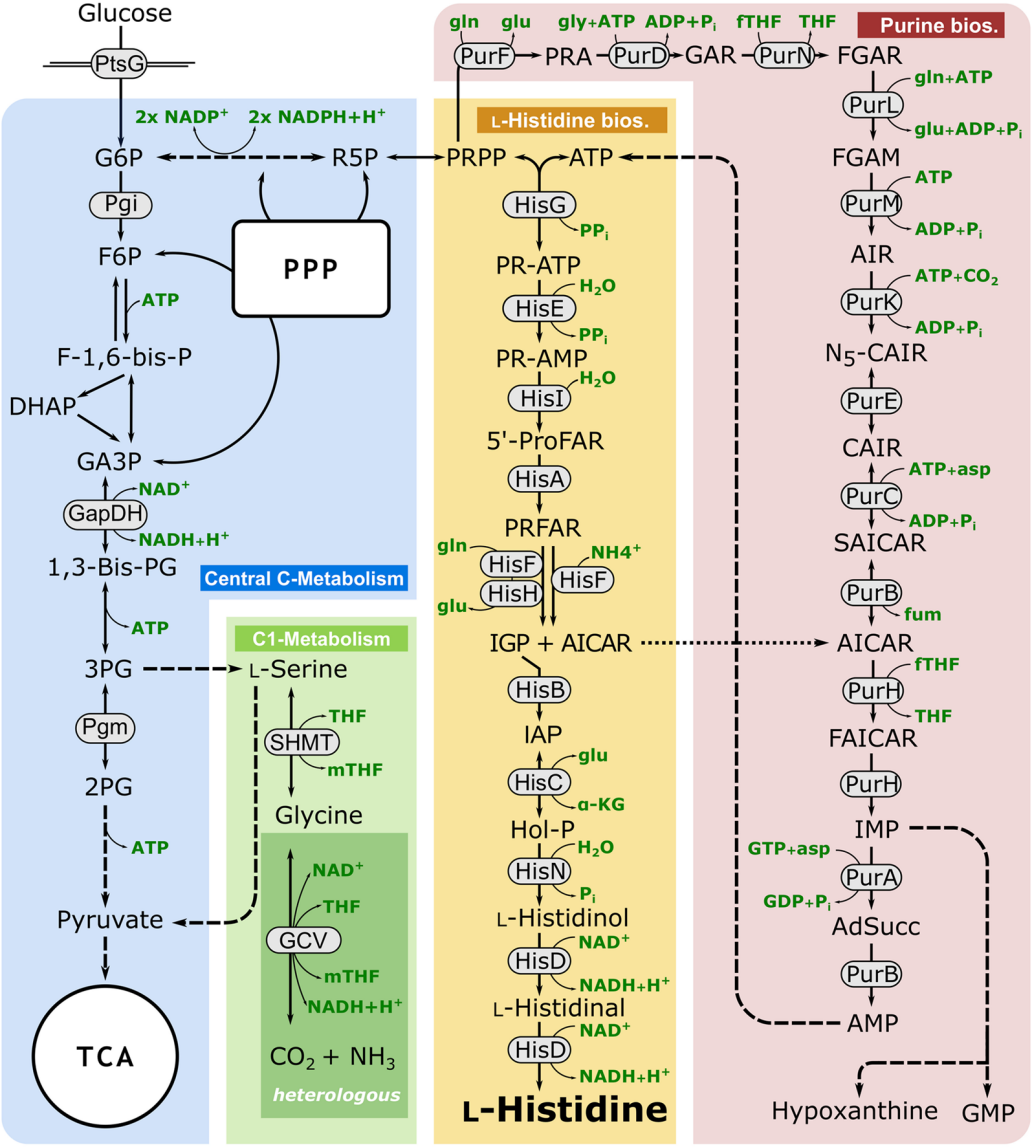
Overview of the modularized metabolism of *C. glutamicum* with focus on the l-histidine biosynthesis (yellow) and the related pathways central carbon metabolism (blue), purine biosynthesis (red), and one carbon metabolism (green). The glycine cleavage (GCV) system is not present in *C. glutamicum* ATCC 13032 and was heterologously produced (dark green). 2PG, 2-phosphoglycerate; 3PG, 3-phosphoglycerate; 5′-ProFAR, 1-(5-phosphoribosyl)-5-[(5-phosphoribosylamino)methylideneamino] imidazole-4 carboxamide; α-KG, α-ketoglutarate; ADP, adenosine diphosphate; AdSucc, adenylosuccinate; AICAR, 1-(5′-phosphoribosyl)-5-amino-4-imidazolecarboxamide; AIR, 5-aminoimidazole ribotide; AMP, adenosine monophosphate; asp; l-aspartate; ATP, adenosine triphosphate; fTHF, 10-formyltetrahydrofolate; F-1,6-bis-P, fructose-1,6-bisphosphate; F6P, fructose 6-phosphate; FAICAR, 5-formamidoimidazole-4-carboxamide ribotide; fGAM, 5′phosphoribosylformylglycineamidine; fGAR, phosphoribosyl-*N*-formylglycineamide; fum, fumarate; G6P, glucose 6-phosphate; GA3P, glyceraldehyde 3-phosphate; GAR, glycineamide ribonucleotide; GCV, glycine cleavage system; gln, l-glutamine; glu, l-glutamate; GMP, guanosine monophosphate; HisA, 5′ProFAR isomerase; HisB, imidazoleglycerol phosphate dehydratase; HisC, histidinol phosphate aminotransferase; HisD, histidinol dehydrogenase; HisE, phosphoribosyl-ATP pyrophosphatase; HisF, synthase subunit of IGP synthase; HisG, ATP phosphoribosyltransferase; HisH, glutaminase subunit of IGP synthase; HisI, phosphoribosyl-AMP cyclohydrolase; HisN, histidinol phosphate phosphatase; Hol-P, l-histidinol phosphate; IAP, imidazole-acetole phosphate; IGP, imidazole-glycerol phosphate; IMP, inosine monophosphate; mTHF, 5,10-methylenetetrahydrofolate; N5-CAIR, 5′-phosphoribosyl-4-carboxy-5-aminoimidazole; NAD+/NADH, oxidized/reduced nicotine amide dinucleotide; NADP+/NADPH, oxidized/reduced nicotine amide dinucleotide phosphate; Pgi, phosphoglucoisomerase; P_i_/PP_i_, inorganic phosphate/diphosphate; Pgm, phosphoglucomutase; PR-AMP, phosphoribosyl-AMP; PR-ATP, phosphoribosyl-ATP; PRA, phosphoribosylamine; PRFAR, 5-[(5-phospho-1-deoxyribulos-1-ylamino)methylideneamino]-1-(5-phosphoribosyl) imidazole-4-carboxamid; PRPP, phosphoribosyl pyrophosphate; PtsG, phosphoenolpyruvate-dependent phosphotransferase system for glucose; PurA, adenylosuccinate synthase; PurB, adenylosuccinate lyase; PurC, phosphoribosylaminoimidazolesuccinocarboxamide synthase; PurD, PRA-glycine ligase; PurE, phosphoribosylaminoimidazole mutase; PurF, amidophosphoribosyltransferase; PurH, bifunctional AICAR formyltransferase/IMP cyclohydrolase; PurK, phosphoribosylaminoimidazole carboxylase; PurL, phosphoribosylformylglycinamide synthase; PurM, phosphoribosylformylglycinamidine cycloligase; PurN, phosphoribosylglycinamide formyltransferase; R5P, ribose 5-phosphate; SAICAR, phosphoribosyl-aminoimidazolesuccinocarboxamide; SHMT, serine hydroxymethyltransferase; TCA, tricarboxylic acid cycle; THF, tetrahydrofolate

Biosynthesis of histidine has been extensively studied, mainly in *Escherichia* *coli* and *Salmonella* *enterica* serovar Typhimurium and several profound reviews are available [1, 28, 96]. Nowadays, histidine biosynthesis is considered fundamentally the same in all living organisms [1]. From a physiological point of view, the biosynthetic pathway of histidine is unique for an amino acid as it is closely entwined with other pathways, such as purine biosynthesis and C_1_ metabolism (Fig. 1). Both the purine and the histidine pathways have the same precursor, phosphoribosyl pyrophosphate (PRPP) (Fig. 1). The second precursor for histidine synthesis is ATP, which is commonly known as energy donor. However, in this unique reaction, the backbone of the ATP molecule is incorporated to give the first intermediate of the histidine pathway. In this reaction catalyzed by ATP phosphoribosyltransferase (HisG), PRPP and ATP are utilized to form phosphoribosyl-ATP, which is further converted in nine enzymatic reactions into histidine (Fig. 1). In the fifth step of the histidine pathway, 5-aminoimidazole-4-carboxamide ribonucleotide (AICAR) is formed and rerouted to the purine biosynthesis, and thus is available to regenerate ATP (Fig. 1). Besides histidine biosynthesis, PRPP is also a precursor for the biosynthetic pathways of pyrimidines, tryptophan, and nicotinamide adenine dinucleotides [45]. The linkage with the C_1_ metabolism manifests in de novo synthesis of purines, where two molecules of 10-formyltetrahydrofolate (fTHF) are required as cofactors (Fig. 1). The major metabolic source for the generation of loaded THF molecules in *C.* *glutamicum* is the reaction of serine hydroxymethyltransferase (SHMT, encoded by *glyA*) that converts l-serine into glycine and simultaneously generates 5,10-methylene-THF (mTHF) from THF [60, 78, 79].

The biosynthesis of histidine in *C.* *glutamicum* consists of ten consecutive enzymatic reactions that are catalyzed by nine enzymes with histidinol dehydrogenase (HisD) being bifunctional [56]. The histidine genes are organized in four operons, comprising *hisD*–*hisC*–*hisB*–*cg2302*–*cg2301*, *hisH*–*hisA*–*impA*–*hisF*–*hisI*–*cg2294*, *cg0911*–*hisN*, and *hisE*–*hisG* (Fig. 7; [47, 56]) and eight of the corresponding histidine genes were described as essential [56]. Besides the feedback inhibition of HisG by histidine [2, 97], transcriptional control of histidine biosynthesis has been shown for the *hisD* operon of *C.* *glutamicum* AS019 to function via a T-box-mediated attenuation mechanism [47, 56]. Analysis of the 5′untranslated region (UTR) of *hisD* in *C.* *glutamicum* ATCC 13032, however, revealed a 103 base pair shorter 5′UTR region and it has been speculated that control of this operon occurs on translational rather than transcriptional level in *C.* *glutamicum* ATCC 13032 [56].

Concerning histidine production, the efforts that have been made with *C.* *glutamicum* are limited to a few examples and classically focused on mutagenesis approaches to increase resistance against histidine analogs and to free HisG from its feedback inhibition [2, 3, 63, 77, 97]. Rational approaches for strain engineering were done by promoter exchange of the *hisD* operon, overexpression of the *hisEG* genes [18], and elimination of feedback inhibition by deleting the C-terminal regulatory domain and mutating the catalytic domain of HisG, combined with *hisEG* overexpression [55]. In addition to modifications in the histidine biosynthesis, decreasing transketolase activity has been shown to improve precursor availability and histidine production [41]. However, a systems metabolic engineering approach to engineer histidine production strains has not yet been conducted. Due to the metabolic complexity of histidine synthesis, we combined rational strain engineering with systems metabolic profiling (SMP) and flux balance analysis (FBA) to identify bottlenecks in the intertwined pathways, and to finally engineer histidine producers with balanced metabolite pools for efficient production.

## Results

### Optimizing the histidine biosynthesis

**Fig. 2:**
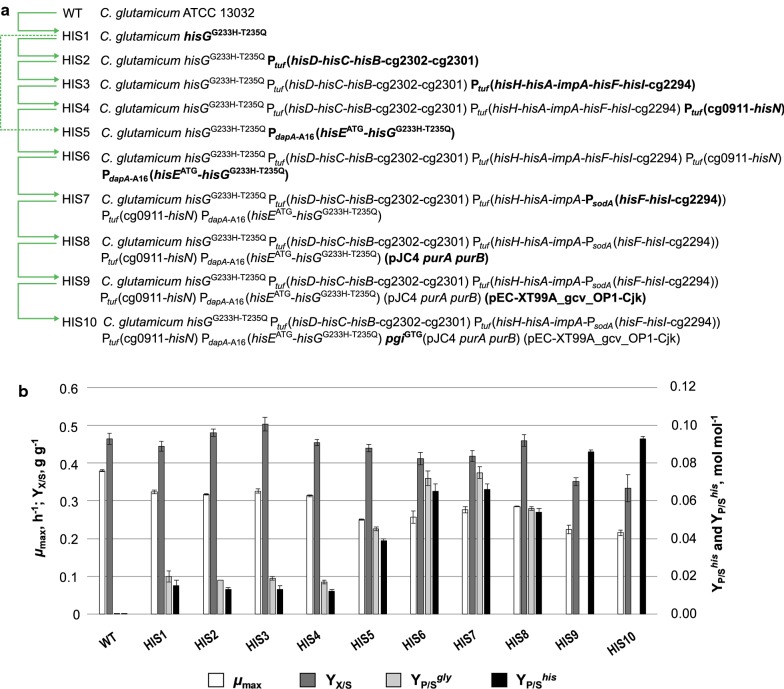
Strain genealogy (**a**) and characteristics (**b**) of the engineered l-histidine-producing strains developed in this work. Arrows indicate direct parentage; new modifications that differentiate the descendant from the progenitor are given in bold (**a**). Characteristics of these strains include maximum growth rate *μ*_max_ (h^−1^) in white, biomass yield per unit substrate *Y*_X/S_ (g g^−1^) in dark gray, and (by)product yield per unit substrate for glycine in gray and l-histidine (mol mol^−1^) in black with the non-producing *C. glutamicum* ATCC 13032 (WT) as reference (**b**). Error bars give standard deviation of at least three independently performed experiments

**Fig. 3:**
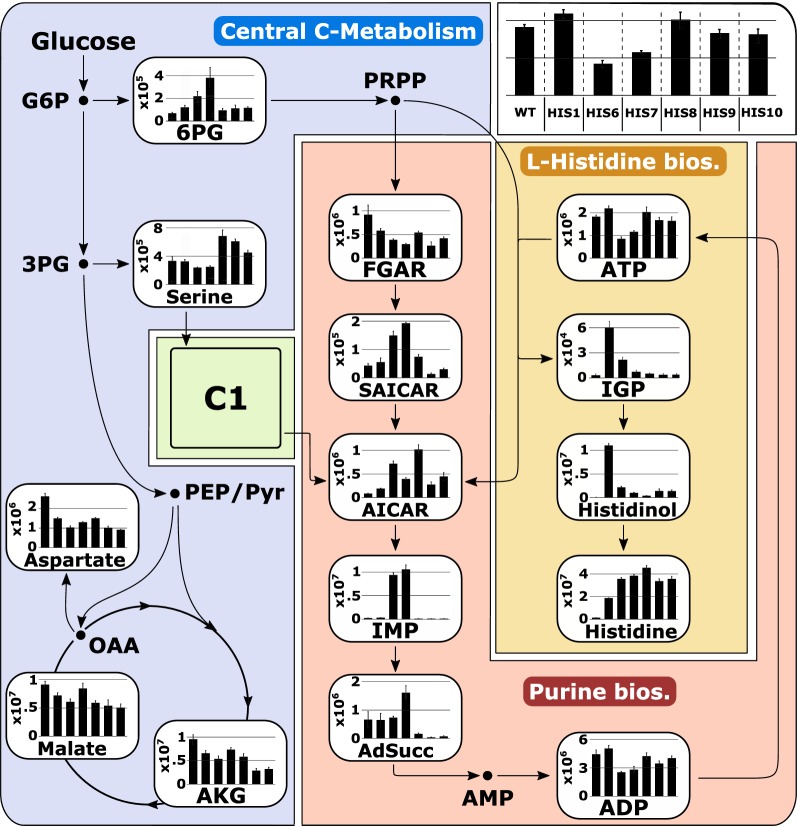
Systems metabolic profiling (SMP) of l-histidine-producer strains *C. glutamicum* HIS1, HIS6–HIS10 and *C. glutamicum* ATCC 13032 (WT). Intracellular peak intensities of each metabolite are given as bar plots. Corresponding strains can be identified per legend in the top right corner. Error bars give standard deviation of three independently performed experiments. Abbreviations can be found in Fig. 1

In the first step, we chromosomally introduced the nucleotide exchanges ggc to cat and acg to cag in *hisG* of *C.* *glutamicum* ATCC 13032 (Fig. 7) to relieve HisG from feedback inhibition, yielding variant HisG^G233H−T235Q^ [77]. The resulting strain *C.* *glutamicum* HIS1 already secreted histidine into the culture supernatant with a product yield per unit substrate (*Y*_P/S_^*his*^) of 0.015 ± 0.003 mol histidine per mol glucose (Fig. 2). Compared to the WT strain, the maximum growth rate *µ*_max_ of *C.* *glutamicum* HIS1 decreased from 0.38 ± 0.01 h^−1^ to 0.32 ± 0.01 h^−1^ and the biomass yield per unit substrate (*Y*_X/S_) remained stable with 0.45 ± 0.01 and 0.46 ± 0.02 g biomass per g substrate, respectively. Beyond this, *C.* *glutamicum* HIS1 produced glycine as main byproduct besides histidine with a *Y*_P/S_^*gly*^ of 0.020 ± 0.003 mol glycine per mol glucose (Fig. 2). To gain a deeper insight into the metabolic state of *C.* *glutamicum* HIS1, systems metabolic profiling (SMP) was performed. Intracellular peak intensities of d-erythro-1-(imidazole-4-yl)glycerol 3-phosphate (IGP) and l-histidinol were 18 and 275 times higher compared to the WT (Fig. 3), respectively. To debottleneck histidine synthesis, we systematically exchanged the native promoters of all four canonical operons containing all histidine biosynthesis genes to stronger ones (Fig. 7), resulting in strains *C.* *glutamicum* HIS2–HIS6. Firstly, the native promoter of the *hisD*–*hisC*–*hisB*–*cg2302*–*cg2301* operon in *C.* *glutamicum* HIS1 was replaced by the promoter of elongation factor TU (P_*tuf*_) and in parallel the 5′UTR region of the *hisD* gene was deleted to eliminate suspected regulatory elements [56] (Fig. 7), which resulted in strain *C.* *glutamicum* HIS2. *C.* *glutamicum* HIS2 showed similar *Y*_P/S_^*his*^ (0.013 ± 0.001 mol histidine per mol glucose), *µ*_max_ (0.32 ± 0.01 h^−1^), *Y*_X/S_ (0.48 ± 0.01 g biomass per g glucose), and *Y*_P/S_^*gly*^ (0.018 mol glycine per mol glucose), compared to *C.* *glutamicum* HIS1. *C.* *glutamicum* HIS3 and HIS4 were generated, by additionally replacing the native promoters of the operons *hisH*–*hisA*–*impA*–*hisF*–*hisI*–*cg2294* and *cg0911*–*hisN* in *C.* *glutamicum* HIS2 with P_*tuf*_ (Fig. 7), respectively. However, the performance of the resulting strains HIS3 and HIS4 remained constant, with *Y*_P/S_^*his*^ of 0.013 ± 0.002 and 0.012 ± 0.001 mol histidine per mol glucose, *µ*_max_ of 0.33 ± 0.01 and 0.31 ± 0.01 h^−1^, *Y*_X/S_ of 0.50 ± 0.02 and 0.45 ± 0.01 g biomass per g glucose, and *Y*_P/S_^*gly*^ of 0.019 ± 0.001 and 0.017 ± 0.001 mol glycine per mol glucose, respectively (Fig. 2). Since we did not succeed in replacing the native promoter of the *hisEG* operon with P_*tuf*_ or the promoter of manganese superoxide dismutase P_*sodA*_ in *C.* *glutamicum* HIS4, we used P_*dapA*–A16_, a mutated variant of the promoter of dihydrodipicolinate synthase (encoded by *dapA*; [88]). Simultaneously, we replaced the native translational start codon GTG of *hisE* by ATG to improve translation efficiency (Fig. 7), which resulted in *C.* *glutamicum* HIS6. The introduced modifications significantly increased histidine production and strain *C.* *glutamicum* HIS6 showed a *Y*_P/S_^*his*^ of 0.065 ± 0.004 mol histidine per mol glucose, which is about 5 times higher compared to the parental strain *C.* *glutamicum* HIS4. Accordingly, the *Y*_P/S_^*gly*^ increased to 0.072 ± 0.004 mol glycine per mol glucose and we observed reduction of 20% and 10% of *µ*_max_ and *Y*_X/S_, respectively (Fig. 2). To evaluate the cumulative effects of the applied modifications, we constructed strain *C.* *glutamicum* HIS5, which carries the HisG^G233H−T235Q^ variant, the exchange of the translational start codon from GTG to ATG of *hisE*, and the replaced native promoter of *hisE* by P_*dapA*–A16_ (Fig. 7). Although, strain *C.* *glutamicum* HIS5 does not possess P_*tuf*_ in front of the remaining three histidine operons, it showed an intermediate *Y*_P/S_^*his*^ of 0.039 ± 0.001 mol histidine per mol glucose which is 160% higher compared to *C.* *glutamicum* HIS1 and 40% lower compared to *C.* *glutamicum* HIS6 (Fig. 2). Thus, the applied modifications in *C.* *glutamicum* HIS6 are cumulatively beneficial for histidine production. Since the *hisH*–*hisA*–*impA*–*hisF*–*hisI*–*cg2294* has a length of > 4000 bps, we finally replaced the internal promoter in front of *hisF* by P_*sodA*_ downstream of an artificial stop codon (Fig. 7). The resulting strain *C.* *glutamicum* HIS7 showed similar characteristics like *C.* *glutamicum* HIS6 (Fig. 2), however the intracellular peak intensities of IGP and l-histidinol were reduced by a factor of 3 and 2, respectively (Fig. 3).

In summary, using SMP, the applied genetic modifications were evaluated and allowed a stepwise increase to a *Y*_P/S_^*his*^ of  0.065 ± 0.004 mol histidine per mol glucose, and readjusted the intracellular concentrations of IGP and l-histidinol to *C.* *glutamicum* WT-like levels (Fig. 3).

### Overexpression of *hisEG* leads to diminished intracellular adenylate levels

**Fig. 4:**
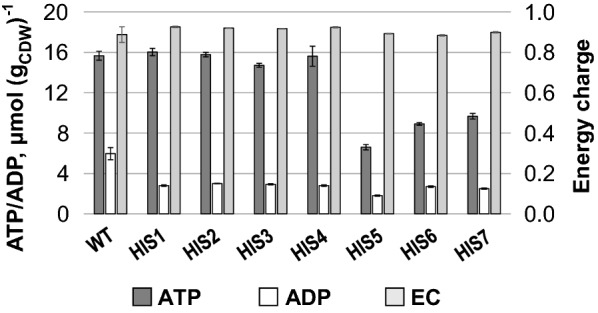
Intracellular ATP and ADP concentrations and the calculated energy charges for strains *C. glutamicum* HIS1–HIS7 with *C. glutamicum* ATCC 13032 (WT) as reference. Intracellular ATP and ADP contents from exponentially growing cells in μmol g_CDW_^−1^ are shown in dark gray and white, respectively. Energy charges are shown as light gray and have been calculated without considering AMP, since AMP concentrations in the histidine producing strains were below the detection limit of the applied HPLC system (see “Methods”). Experiments were performed in at least triplicates and standard deviations are given as error bars

Since histidine and purine biosynthesis are closely interlinked (Fig. 1), we determined the intracellular adenylate concentrations in strains *C.* *glutamicum* HIS1–HIS7 and calculated adenylate energy charges (ECs), as a measure for the energetic state of the strains. Interestingly, we found relatively stable ECs in all engineered strains with values between 0.88 and 0.92, which are comparable to the WT strain, with an EC of 0.89 ± 0.04 (Fig. 4). Despite the stable ECs, the absolute concentrations of ATP, ADP, and AMP were strongly affected (Fig. 4). The WT strain showed intracellular purine concentrations of 15.7 ± 0.4 µmol ATP g_CDW_^−1^, 6.0 ± 0.6 µmol ADP g_CDW_^−1^, and 2.1 ± 0.8 µmol AMP g_CDW_^−1^ during exponential growth phase (AMP not shown). According to their intracellular ATP and ADP concentrations, strains *C.* *glutamicum* HIS1–HIS7 can be clustered into two groups. The first group (*C.* *glutamicum* HIS1–HIS4) exhibited solely diminished ADP levels, showing about half of the concentration of the WT strain; whereas ATP concentrations remained stable. The second group (*C.* *glutamicum* HIS5–HIS7) exhibited diminished ADP and also diminished ATP concentrations with values ranging from 6.6 to 9.7 µmol ATP g_CDW_^−1^ and 1.8–2.5 µmol ADP g_CDW_^−1^. Strikingly, ATP concentrations were only affected in strains where the native promoter of the *hisEG* operon was exchanged by P_*dapA*–A16_. As described in the materials and methods part, intracellular AMP concentrations in the modified strains were below the detection limit of the applied method, and it, thus, can be concluded that AMP concentrations in strains *C.* *glutamicum* HIS1–HIS7 were significantly below the concentrations of the WT strain.

### Energy engineering to readjust ATP levels for histidine production

**Fig. 5:**
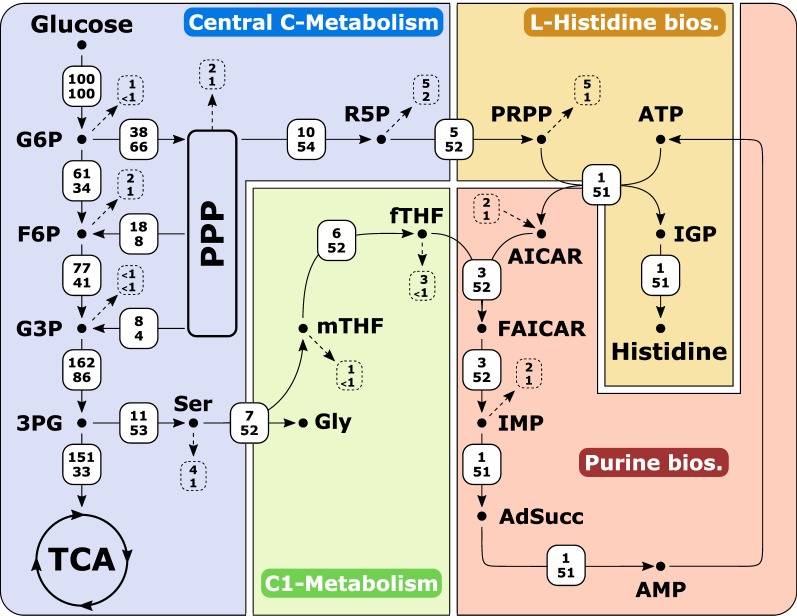
Flux distribution of WT-like growth (upper number) and growth coupled l-histidine production (lower number) in *C. glutamicum*. All fluxes are given in percent (%) of glucose uptake rate, which was set to 3.94 mmol gCDW^−1^ h^−1^ [12] for both simulations. Additional constraint for growth coupled l-histidine production: *μ* = 0.1 h^−1^. Dotted lines show respective flux to biomass. For abbreviations see Fig. 1

After optimizing the histidine biosynthesis and investigating intracellular adenylate concentrations, we performed flux balance analysis (FBA) to gain a holistic overview and further indications to improve histidine production with *C.* *glutamicum* (Fig. 5). Two different FBAs resembling WT-like growth and growth-coupled histidine production were performed, which showed that in addition to an increased flux through the histidine biosynthesis pathway, an equimolar supply of ATP is a prerequisite for efficient histidine production. Therefore, an unphysiologically high flux from AICAR to ATP has to be accomplished (Fig. 5). To identify bottlenecks in this ATP regeneration cascade, we performed further SMP, and found that in *C.* *glutamicum*, HIS7 strongly increased intracellular peak intensities of inosine monophosphate (IMP) and adenylosuccinate, which were 43- and twofold higher compared to the WT (Fig. 3). The strongly increased IMP level indicated a limitation on the level of adenylosuccinate synthetase (PurA) and/or adenylosuccinate lyase (PurB), which catalyze the subsequent reactions from IMP over adenylosuccinate to AMP, respectively. To improve the ATP regeneration capacity (designated as energy engineering), we overexpressed the native *purA* and *purB* genes from the plasmid pJC4 under control of P_*tuf*_ in *C.* *glutamicum* HIS7. Compared to the parental strain *C.* *glutamicum* HIS7, the plasmid-carrying derivative *C.* *glutamicum* HIS8 had a 17% lower *Y*_P/S_^*his*^ of 0.054 ± 0.002 mol histidine per mol glucose. The growth rate remained stable with 0.29 ± 0.01 h^−1^ and the *Y*_X/S_ increased slightly to 0.46 ± 0.02 g biomass per g glucose. Concomitant with the reduced histidine yield, the glycine yield decreased to 0.056 ± 0.001 mol glycine per mol glucose (Fig. 2). However, SMP of *C.* *glutamicum* HIS8 showed that upon introduction of pJC4*purApurB,* the intracellular levels of IMP and adenylosuccinate were readjusted to WT-like levels. Additionally, intracellular peak intensities of ATP and ADP were restored from 64 and 63% (*C.* *glutamicum* HIS7) to 111% and 96% of the WT level in *C.* *glutamicum* HIS8 (Fig. 3). Taken together, the applied energy engineering approach identified the requirement of an unphysiologically high ATP regeneration capacity for histidine production which can be accounted for by overexpression of *purA* and *purB*. Although this modification readjusted the intracellular purine pool, the *Y*_P/S_^*his*^ was not improved indicating further obstacles that must be overcome.

### C_1_ supply is a further bottleneck for histidine production

**Fig. 6:**
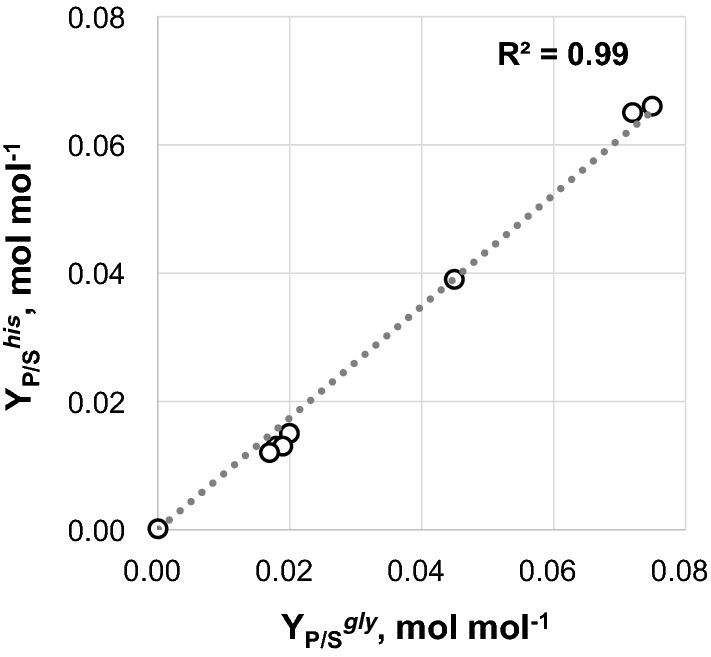
Correlation of the product substrate yields (mol mol^−1^) for histidine (*Y*_P/S_^*his*^) and glycine (*Y*_P/S_^*gly*^) of strains *C. glutamicum* HIS1–HIS7 including values for *C. glutamicum* ATCC 13032. Given product substrate yields (open circles) are mean values calculated from at least three independent experiments

**Fig. 7:**
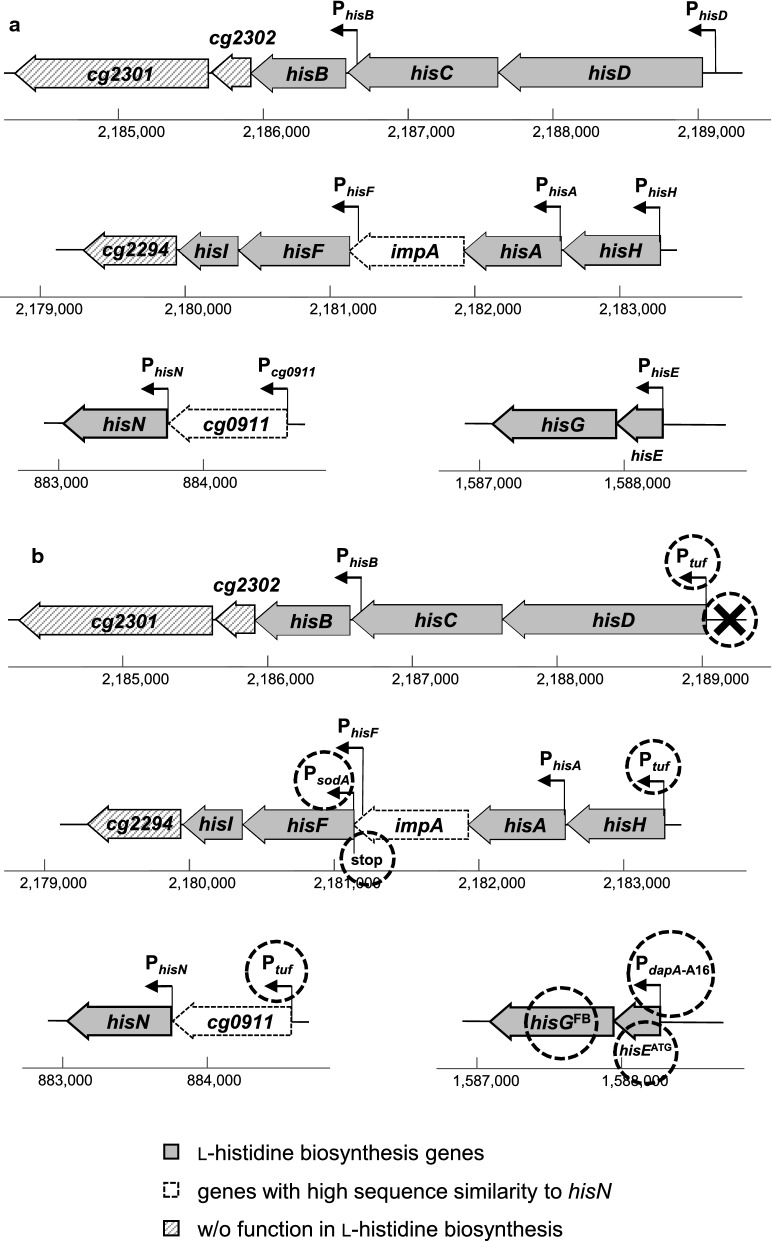
Schematic overview of the structure of the four operons containing l-histidine biosynthesis genes before (**a**) and after (**b**) genetic engineering applied in this study. Dashed circles show where modifications were introduced. The cross marks deletion of the promoter region. Modified after [56]

For incorporation of the two carbonyl groups into the carbon skeleton of purines, the de novo biosynthesis requires two molecules of fTHF, which are provided as mTHF by the conversion of l-serine into glycine catalyzed by serine hydroxymethyltransferase (SHMT; Fig. 1). This is reflected in the conducted FBAs, yielding an equimolar flux into C_1_ metabolism and histidine synthesis (Fig. 5). Since the surplus of glycine cannot be further degraded or rerouted to the central metabolism of *C.* *glutamicum*, all strains of the so far engineered strain genealogy produced glycine as the main byproduct being inevitably present in equimolar amounts to histidine (Fig. 6). Although, overexpression of *purA* and *purB* reduced the intracellular peak intensities of IMP and adenylosuccinate to WT levels, we observed that the intracellular AICAR peak intensity was not reduced but even increased (2.6-fold) in *C.* *glutamicum* HIS8 compared to *C.* *glutamicum* HIS7 (Fig. 3). This result pointed to a fTHF limitation of the bifunctional AICAR formyltransferase/IMP cyclohydrolase (PurH, Fig. 1), which might negatively affect the flux in the upper part of histidine synthesis. Since the glycine cleavage (GCV) system converts glycine into CO_2_, ammonia, and simultaneously generates mTHF from THF (Fig. 1), we heterologously expressed the GCV system from *C.* *jeikeium* on plasmid pEC-XT99A_gcv_OP1-Cjk in strain *C.* *glutamicum* HIS8, resulting in *C.* *glutamicum* HIS9. This modification was expected to reduce glycine accumulation accompanied by an improvement of the supply with loaded THF molecules. *C.* *glutamicum* HIS9 showed a *µ*_max_ of 0.22 ± 0.01 h^−1^, a *Y*_X/S_ of 0.35 ± 0.01 g biomass per g glucose and in contrast to *C.* *glutamicum* HIS8 did not secret glycine into the supernatant (Fig. 2). Furthermore, the *Y*_P/S_^*his*^ in *C.* *glutamicum* HIS9 increased by 59% to 0.086 ± 0.001 mol histidine per mol glucose and the peak intensities of AICAR decreased by 73% compared to *C.* *glutamicum* HIS8 (Figs. 2, 3). In summary, the expression of a functional GCV system eliminated glycine as byproduct, increased the fTHF availability and, in combination with *purA* and *purB* overexpression, improved histidine production with *C.* *glutamicum*.

### Engineering the glycolysis–PPP split ratio

To achieve an optimal flux distribution for histidine production with *C.* *glutamicum*, the FBA predicted to increase the carbon flux towards the pentose phosphate pathway (PPP) by 74% compared to the WT flux (Fig. 5). Thus, *C.* *glutamicum* HIS9 with optimized energy metabolism and enhanced C_1_ supply was further modified to reroute carbon from glycolysis to the PPP by changing the native translational start codon ATG of the *pgi* gene, encoding the glucose 6-phosphate isomerase (Pgi), to the weaker GTG. The constructed strain *C.* *glutamicum* HIS10 showed a similar *µ*_max_ and *Y*_X/S_, however, a *Y*_P/S_^*his*^ of 0.093 ± 0.001 mol histidine per mol glucose which is 8% higher compared to *C.* *glutamicum* HIS9 (Fig. 2).

## Discussion

Histidine is an attractive amino acid for various applications in the feed and medical sector [25, 87, 90, 93] and in 2003, the production by fermentation was estimated to be 400 t histidine per year [41]. Most efficient producer strains described in literature have been obtained by classical mutagenesis and show maximal *Y*_P/S_^*his*^ values of about 0.15–0.20 g histidine per g substrate [9, 63], which is about 2.5-fold lower than the maximum theoretical product yield of 0.44 g per g achieved at *μ*_*max*_ = 0.1 h^−1^ in growth-coupled manner (Fig. 5). Associated with the applied modifications in the histidine biosynthesis and connected pathways, *µ*_max_ strongly decreased to a minimum of 0.22 ± 0.01 h^−1^ in *C.* *glutamicum* HIS9 and HIS10 (equals about 58% of WT *µ*_max_) which mostly can be attributed to the overexpression of the modified *hisEG* genes (Fig. 2). Both *µ* and *Y*_P/S_^*his*^, which is about 18% of the theoretical maximum, are crucial factors for industrial-scale application and therefore have to be optimized in further studies. So far, few studies provided knowledge for the targeted optimization of *C.* *glutamicum* as a histidine overproducer. As such, the deregulation of the biosynthesis and the improved precursor availability [41, 55, 63, 97] have been investigated. The moderate success to develop efficient production strains might be attributed to the demanding biosynthesis of histidine reflected by its tight connection to energy metabolism (Fig. 1). Therefore, to gain a more holistic understanding of the metabolic limitations for histidine production, we performed a modularized metabolic engineering approach, including flux balance analysis and LC/MS QToF-based systems metabolic profiling (SMP). Especially, the applied untargeted metabolomics workflow proofed an effective tool to monitor intracellular peak intensities of key metabolites in the engineered strain genealogy. The introduced modifications in the histidine biosynthesis enabled histidine overproduction (Fig. 2) and were shown for *C.* *glutamicum* HIS7 to maintain WT-like levels of the intermediates IGP and l-histidinol (Fig. 3). In contrast, the intracellular peak intensities of histidine increased stepwise with *C.* *glutamicum* HIS8 showing a 33 times higher level compared to the WT, indicating an export limitation. However, to our knowledge, no export system for histidine has been identified so far in *C.* *glutamicum,* whereas the gene product of *cg1305* was proposed to be involved in histidine import [56]. If the prevention of re-import might be beneficial for histidine production has to be verified in future experiments.

The quantification of intracellular ATP and ADP concentrations in strains *C.* *glutamicum* HIS1–HIS7 (Fig. 4) showed that particularly the overexpression of *hisEG* (presumably the mutated *hisG*, since it encodes the first enzyme in the biosynthesis pathway, which catalyzes the covalent binding of ATP to PRPP) in strain *C.* *glutamicum* HIS5 drained ATP efficiently into histidine biosynthesis and led to strongly diminished purine concentrations. Interestingly, the perturbation of the energy metabolism did not manifest in an altered energy charge itself but was disclosed by consistent reduction of the ATP and ADP pools (Fig. 4), which underpins the relevance of a balanced energy state in the regulatory hierarchy of the cell [4, 5]. The energy charge of the adenylate pool as a regulatory parameter. Interaction with feedback modifiers. Biochemistry [4, 92]. The applied FBA already pointed to the requirement of a high ATP regeneration capacity of the cell for efficient histidine production (Fig. 5) and SMP finally hinted to PurA and/or PurB as the limiting step(s) by the observed strong increase of the IMP and adenylosuccinate levels in *C.* *glutamicum* HIS 6 and HIS7, compared to the WT (Fig. 3). Indeed, overexpression of *purA* and *purB* not only reduced the intracellular peak intensities of IMP and adenylosuccinate but also readjusted the ADP and ATP levels to WT level demonstrating that the natural capacity of the cell is not suited to regenerate ATP on top of the growth demands.

Recently, *E.* *coli* has also been engineered for histidine production and the observed intracellular accumulation of AICAR was overcome by introduction of an additional copy of *purA* into the chromosome [62]. Interestingly, although overexpression of *purA* and *purB* in our strains almost readjusted the levels of IMP, adenylosuccinate, ADP, and ATP, SMP revealed still increased peak intensities for AICAR in all histidine producing strains compared to the WT (Fig. 3) indicating a different regulatory pattern in *C.* *glutamicum* compared to *E.* *coli*. Since overexpression of *purA* and *purB* positively impacted the energy state of the cell but did not improve the histidine yield, we speculated that the increased intracellular AICAR levels feedback on the upper part of the histidine synthesis pathway and indicate another bottleneck in the metabolism of *C.* *glutamicum*. In accordance, Malykh et al. [62] recently suggested in *E.* *coli* a competitive inhibitory influence of AICAR on HisG. Furthermore, it has been shown for *E.* *coli* that upon folate limitation, AICAR accumulates and binds to a specific riboswitch, which negatively controls expression of purine genes [50]. Likewise, in *C. glutamicum,* the conversion of AICAR by PurH is fTHF dependent (Fig. 1), and consequently we speculated about a C_1_ limitation for histidine production, which was supported by the results of FBA, proposing a required high flux into the C_1_ metabolism (Fig. 5). Furthermore, strains *C.* *glutamicum* HIS1–HIS8 secreted glycine as inevitable equimolar byproduct to histidine, which has also been observed for other histidine producing mutants of *C.* *glutamicum* and *Brevibacterium* *flavum* [19, 44].

The required fTHF for purine biosynthesis is supplied by the reaction of the SHMT, converting l-serine into glycine, thereby generating mTHF, which might be further converted into fTHF [29, 39, 78, 79]. Unfortunately, the various THF species of the C_1_ metabolism are not accessible with the applied analytical system, due to low pool sizes caused by interconversion, polyglutamylation, and degradation [61]. However, the C_1_ cycle is a complex network of several oxidized/reduced forms of C_1_ units with THF as carrier molecule and has been investigated before for l-methionine- and l-serine-overproducing *C.* *glutamicum* strains [15, 32, 54, 81]. To overcome the proposed C_1_ limitation, we expressed the GCV system from *C.* *jeikeium* in *C.* *glutamicum* HIS8, which already overexpresses the *purA* and *purB* genes, and in fact observed the disappearance of glycine as byproduct (Fig. 2), a significant reduction of the AICAR pool (Fig. 3), and a significantly increased *Y*_P/S_^*his*^ (Fig. 2). In a recent approach, a GCV system from *E.* *coli* has been heterologously produced in *C.* *glutamicum*, where it enabled increased l-serine accumulation in a *glyA* attenuated strain, by generating improved amounts of C_1_ units for incorporation in the purine biosynthesis [99]. Consistent with these data, the GCV system from *C.* *jeikeium* seems to be able to (partly) satisfy the need for loaded THF molecules in histidine-producing *C.* *glutamicum*. However, the remaining elevated AICAR levels in *C.* *glutamicum* HIS9 and HIS10 (Fig. 3) either indicate an even higher demand for fTHF or point to limiting AICAR formyltransferase/IMP cyclohydrolase activity, which might be overcome by overexpression of *purH*.

## Conclusions

Taken together, the applied interplay of strain engineering, systems metabolic profiling, and flux balance analysis yielded a comprehensive view on the complex metabolic network of histidine biosynthesis. Energy engineering identified and reinforced the intrinsically low ATP regeneration capacity to maintain the balanced energy state of the cell. However, to utilize the readjusted ATP levels for histidine production, it is essential to provide sufficient C_1_ units avoiding the accumulation of AICAR, which seems to be a potent effector molecule to control the entry flux into histidine biosynthesis.

## Methods

### Strains and plasmids

All strains and plasmids used in this study are listed in Table 1. Oligonucleotides are given in Table 2.

**Table 1:** Strains and plasmids that were used in this study

Strain or plasmid	Relevant characteristic(s)	Source or references
Strains		
*E. coli* DH5α	F-ϕ80*lacZ*ΔM15 Δ(*lacZYA*-*argF*)U169 *endA1 recA1 hsdR17* (r_K_^−^ m_K_^+^) *supE44 thi*-*1 gyrA96 relA1 phoA*	Hanahan [31]
*E. coli* K-12 MG1655	Wild-type strain DSM 18039; F-, λ-, *ilvG*-, *rfb*-50, *rph*-1	German collection of microorganisms and cell cultures
*C. glutamicum* WT	Wild-type strain ATCC 13032	American type culture collection
*C. jeikeum* K411	Wild-type strain	Tauch et al. [84]
*C. glutamicum* HIS1	*C. glutamicum* WT with the feedback inhibition released variant HisG^G233H−T235Q^ (corresponding nucleotide exchanges: ggc742cat, acg748cag)	This work
*C. glutamicum* HIS2	*C. glutamicum* HIS1 with additional implementation of P_*tuf*_ in front of the operon *hisD*–*hisC*–*hisB*–*cg2302*–*cg2301*	This work
*C. glutamicum* HIS3	*C. glutamicum* HIS2 with additional implementation of P_*tuf*_ in front of the operon *hisH*–*hisA*–*impA*–*hisF*–*hisI*–*cg2294*	This work
*C. glutamicum* HIS4	*C. glutamicum* HIS3 with additional implementation of P_*tuf*_ in front of the operon *cg0911*–*hisN*	This work
*C. glutamicum* HIS5	*C. glutamicum* HIS1 with additional implementation of P_*dapA*–A16_ in front of the operon *hisE*–*hisG*, additional exchange of the translational start codon from the native GTG to ATG for *hisE*	This work
*C. glutamicum* HIS6	*C. glutamicum* HIS4 with additional implementation of P_*dapA*–A16_ in front of the operon *hisE*–*hisG*, additional exchange of the translational start codon from the native GTG to ATG for *hisE*	This work
*C. glutamicum* HIS7	*C. glutamicum* HIS6 with additional implementation of P_*sodA*_ in front of gene *hisF*	This work
*C. glutamicum* HIS8	*C. glutamicum* HIS7 containing pJC4 *purA purB*	This work
*C. glutamicum* HIS9	*C. glutamicum* HIS8 containing pEC-XT99A_gcv_OP1-Cjk	This work
*C. glutamicum* HIS10	*C. glutamicum* HIS9 with additional exchange of the translational start codon from ATG to GTG	This work
Plasmids		
pK19*mobsacB*	Km^r^, mobilizable (*oriT*), *oriV*	Schäfer et al. [75]
pK18*mobsacB* P*aceE dapA*-A16	pK18*mobsacB* carrying the *dapA*-A16 promoter	Buchholz et al. [16]
pK19*mobsacB hisG*^FB^	pK19*mobsacB* carrying the nucleotide sequence of a modified *hisG* variant that encodes HisG with amino acid exchanges G233H and T235Q (corresponding nucleotide exchanges: ggc742cat, acg748cag)	This work
pK19*mobsacB hisD*-P_*tuf*_	pK19*mobsacB* carrying promoter exchange to P_*tuf*_ for operon *hisD*–*hisC*–*hisB*–*cg2302*–*cg2301*	This work
pK19*mobsacB hisH*-P_*tuf*_	pK19*mobsacB* carrying promoter exchange to P_*tuf*_ for operon *hisH*–*hisA*–*impA*–*hisF*–*hisI*–	This work
pK19*mobsacB hisN*-P_*tuf*_	pK19*mobsacB* carrying promoter exchange to P_*tuf*_ for operon *cg0911*–*hisN*	This work
pK19*mobsacB hisE*-P_*dapA*–A16_	pK19*mobsacB* carrying promoter exchange to P_*dapA*_–_A16_ for operon *hisE*–*hisG* and an exchange of the translational start codon from the native GTG to ATG for *hisE*	This work
pJC4	Km^r^	Cordes et al. [20]
pJC4 *purA purB*	pJC4 carrying genes *purA* and *purB* from *C. glutamicum* ATCC 13032 under control of P_*tuf*_ and T_*rrnB*_	This work
pEC-XT99A	IPTG-inducible overexpression plasmid	Kirchner and Tauch [51]
pEC-XT99A_gcv_OP1-Cjk	IPTG-inducible overexpression plasmid for genes *gcvP*, *gcvT*, *gcvH*, *lipA*, and *lipB* from *C. jeikeium* xxx	(A. Hüser, Evonik Nutrition & Care GmbH)

### Media and cultivation conditions

*Escherichia coli* DH5α was used as cloning host and was grown aerobically in 2 × YT complex medium [74] in a 5-mL glass test tube culture at 37 °C on a rotary shaker at 120 rpm. Precultures of *C.* *glutamicum* strains were prepared by thawing a glycerol stock (30% w v^−1^ glycerol) and streaking cell solution on a 2 × YT agar plate which was incubated at 30 °C for 2 days. A single colony of the respective strain was then used to inoculate 5 mL of 2 × YT complex medium in a glass test tube, which was incubated at 30 °C on a rotary shaker at 120 rpm for 6–8 h. The complete suspension of the glass test tube was transferred into 50 mL of 2 × YT medium in a 500-mL baffled shaking flask, which was incubated at 30 °C on a rotary shaker at 120 rpm overnight. To inoculate the main culture, cells were harvested by centrifugation (4500×*g*, 10 min, 4 °C), the pellet was resuspended in 0.9% w v^−1^ NaCl solution and used to inoculate CGXII minimal medium to an optical density at 600 nm (OD_600_) of about 2.5. The CGXII minimal medium [24] is composed of 20 g (NH_4_)_2_SO_4_ L^−1^, 5 g urea L^−1^, 21 g 3-morpholinopropanesulfonic acid (MOPS) L^−1^, 1 g K_2_HPO_4_ L^−1^, 1 g KH_2_PO_4_ L^−1^, 0.25 g MgSO_4_ L^−1^, 0.01 g CaCl_2_ L^−1^. The pH value of the medium was adjusted to 7.4 with 5 M KOH before autoclaving. Then, 16.4 mg FeSO_4_ × 7 H_2_O L^−1^, 10 mg MnSO_4_ × H_2_O L^−1^, 0.2 mg CuSO_4_ L^−1^, 1 mg ZnSO_4_ × 7 H_2_O L^−1^, 0.02 mg NiCl_2_ × 6 H_2_O L^−1^, 0.2 mg biotin L^−1^ were added sterilely. Standard cultivations in shaking flasks contained 10 g glucose L^−1^ as carbon source. For cultivations of strains bearing plasmid pJC4, 50-µg kanamycin mL^−1^ was added. For strains harboring plasmid pJC4 and pEC-XT99A, the kanamycin concentration was decreased to 12.5 µg mL^−1^ and 2.5-µg tetracycline mL^−1^ was added. The expression from P_*trc*_ in pEC-XT99A_gcv_OP1-Cjk was induced by adding 1 mM isopropyl β-d-1-thiogalactopyranoside (IPTG) at the cultivation start. OD_600_ was measured with a photometer (Ultrospec 10 Cell Density Meter, GE Healthcare Company, Little Chalfont, UK). The cell dry weight (CDW in g L^−1^) was calculated using the correlation CDW = OD_600_ × 0.21 g L^−1^.

### Determination of *µ*_max_, *Y*_X/S_, and *Y*_P/S_

The maximal growth rate *µ*_max_ was determined by linear regression of ln(OD_600_), which was plotted against the cultivation time in h during the exponential growth phase of the respective strain. The biomass yield per unit substrate *Y*_X/S_ in g g^−1^ was determined by linear regression of the biomass concentration in g L^−1^, which was plotted against the corresponding glucose concentration in g L^−1^. The product yields per unit substrate for histidine (*Y*_P/S_^*his*^) and glycine (*Y*_P/S_^*gly*^) in mol mol^−1^ were determined by dividing the product concentration (histidine) in mol L^−1^ and byproduct concentration (glycine) in mol L^−1^ after 24 h by the corresponding initial substrate concentration in mol L^−1^ at 0 h, respectively.

### Genetic manipulation

Molecular cloning methods, such as PCR and DNA restriction, were carried out according to [74]. Plasmids were isolated with E.Z.N.A. Plasmid Mini Kit I (Omega Bio-tek Inc., Norcross, USA) and PCR fragments were purified with NucleoSpin Gel and PCR Clean-up Kit (Macherey–Nagel GmbH & Co. KG, Düren, Germany) according to the manufacturer’s instructions. Electrocompetent cells of *E.* *coli* and *C.* *glutamicum* were prepared as described before [22, 85]. Constructed plasmids were transformed into *E.* *coli* according to [22], and into *C.* *glutamicum* with a subsequent heat shock after transformation for 6 min at 46 °C according to Rest et al. [73]. Plasmids were transformed into electrocompetent *E.* *coli* and *C.* *glutamicum* strains with an Eporator (Eppendorf AG, Hamburg, Germany) at 2.5 kV with 600 Ω resistance. Enzymes for recombinant DNA work were obtained from Thermo Scientific Inc. (Darmstadt, Germany) and oligonucleotides were synthesized by biomers.net GmbH (Ulm, Germany, listed in Table 2).

Promoter exchanges and nucleotide substitutions were performed via a two-step homologous recombination by applying the respective pK19*mobsacB* derivative [75]. The plasmid to exchange the native HisG variant with the feedback-released HisG^G233H–T235Q^ [77] was implemented into *C.* *glutamicum* ATCC 13032 by amplifying the flanking genomic regions of *hisG* up- and downstream of the mutations with primer pairs hisG1/hisG2 and hisG3/hisG4 (hisG2 and hisG3 harbor the exchanges). Both polymerase chain reaction (PCR) products were used as templates in a SOEing PCR [38] with primer pair hisG1/hisG4. The SOEing product and pK19*mobsacB* were digested with *Bam*HI and fused together in a ligation reaction to give pK19*mobsacB* *hisG*^FB^. This plasmid was then transformed into *E.* *coli* DH5α, isolated, and its sequence integrity was verified by DNA sequencing with primers pK19-fw and pK19-rev (GATC Biotech AG, Constance, Germany). The verified plasmid was then transformed into *C.* *glutamicum* ATCC 13032. Applying the method described by Schäfer et al. [75], the native *hisG* sequence was replaced via homologous recombination (double crossover) by the mutated *hisG* sequence leading to amino acid exchanges G233H and T235Q. The screening of the *C.* *glutamicum* HIS1 mutants was done on 2 × YT agar plates containing 10% (w v^−1^) sucrose. For verification of the nucleotide exchanges, a PCR with primer pair hisG1/hisG4 was performed and sent for sequencing with primer hisGseq. To construct plasmids for the promoter exchanges in front of the operons containing histidine biosynthesis genes (*C.* *glutamicum* HIS2, HIS3, HIS4, and HIS6), the flanking regions of the respective promoter were amplified. For the exchange of the native promoter of the operon *hisD*–*hisC*–*hisB*–*cg2302*–*cg2301* with the strong promoter of the gene *tuf*, encoding the elongation factor TU, the flanking regions were amplified with primer pairs hisD1/hisD2 and hisD3/hisD4. The products of both PCRs were used as templates in a SOEing PCR with primer pair hisD1/hisD4, and the SOEing product and the plasmid pK19*mobsacB* were digested with *Bam*HI and *Hin*dIII and ligated together to give an intermediate plasmid. This plasmid was transformed into *E.* *coli* DH5α, isolated and sent for sequencing with primers pK19-fw and pK19-rev. In the next step, P_*tuf*_ was amplified with primer pair tuf1/tuf2, and the product and the intermediate plasmid were digested with *Nde*I and *Nsi*I. Both were ligated to give plasmid pK19*mobsacB* *hisD*-P_*tuf*_, which was transformed into *E.* *coli* DH5α, isolated and sent for sequencing with primers pK19-fw and pK19-rev. The verified pK19*mobsacB* *hisD*-P_*tuf*_ was transformed into *C.* *glutamicum* HIS1 and exchange of the native promoter region with the P_*tuf*_ promoter was performed as has been described above yielding *C.* *glutamicum* HIS2. The respective region was amplified with primer pair hisD1/Ptuf2 and sequenced with primer hisD1. The plasmids for exchanging the native promoter with P_*tuf*_ for operons *hisH*–*hisA*–*impA*–*hisF*–*hisI*–*cg2294* and *cg0911*–*hisN* were constructed accordingly. Primer pairs hisH1/hisH2 and hisH3/hisH4 and hisN1/hisN2 and hisN3/hisN4 were used to amplify the flanking regions, respectively. After SOEing PCR, digestion, and ligation, plasmids were transformed into *E.* *coli* DH5α and prepared. In further steps, the mentioned plasmids were digested with *Nde*I and *Nsi*I and fused with the P_*tuf*_ region. After sequencing, pK19*mobsacB* *hisH*–P_*tuf*_ was implemented in *C.* *glutamicum* HIS2 to yield *C.* *glutamicum* HIS3. The sequence was verified with primers hisH1, hisH4, tuf1, and tuf2. *C.* *glutamicum* HIS3 served as basis for implementing P_*tuf*_ in front of *cg0911*–*hisN* using pK19*mobsacB hisN*–P_*tuf*_ to yield *C.* *glutamicum* HIS4. This strain was verified with primers hisN1, hisN4, tuf1, and tuf2. Since we were not able to implement P_*tuf*_ in front of the *hisE*–*hisG* operon in *C.* *glutamicum* HIS4, we instead used P_*dapA*–A16_ [88], a modified version of the promoter of dihydrodipicolinate synthase, which was amplified with primer pair dapA1/dapA2 from pK18*mobsacB* P*aceE* *dapA*-A16 [16]. The flanking regions of the *hisE*–*hisG* promoter were amplified with primer pairs hisE1/hisE2 and hisE3/hisE4, a SOEing PCR was prepared with primer pair hisE1/hisE4. This product and pK19*mobsacB* were digested with *Bam*HI and *Hin*dIII and ligated. P_*dapA*-A16_ and this plasmid were digested with *Nde*I and *Nsi*I and ligated. Hence, on the basis of *C.* *glutamicum* HIS4, *C.* *glutamicum* HIS6 was created and verified with primers hisE1, hisE4, dapA1, and dapA2. *C.* *glutamicum* HIS5 was created by implementing P_*dapA*–A16_ in *C.* *glutamicum* HIS1. To exchange the internal promoter of *hisF* in the operon *hisH*–*hisA*–*impA*–*hisF*–*hisI*–*cg2294* with the promoter of manganese superoxide dismutase (encoded by *sodA*), flanking regions and the promoter were amplified with primer pairs hisF1/hisF2, sodA1/sodA2, and hisF3/hisF4 and an additional stop codon (TAA) was integrated upstream of *hisF*. The SOEing PCR (with all three products as template and primer pair hisF1/hisF4) and pK19*mobsacB* were cut with *Hin*dIII and *Bam*HI and ligated together. Integration of P_*sodA*_ in front of *hisF* in strain *C.* *glutamicum* HIS6 yielded *C.* *glutamicum* HIS7, which was verified with primers hisF4 and hisFseq.

On the basis of plasmid pJC4 [20], we constructed pJC4 *purA* *purB* by amplifying P_*tuf*_, *purA*, and *purB* with primer pairs tuf2_1/tuf2_2, purA1/purA2, and purB1/purB2 from the *C.* *glutamicum* genome. Furthermore, primer pair rrnB1/rrnB2 was used to amplify the T_*rrnB*_ terminator region of the *rrnB* operon from the *E.* *coli* K-12 MG1655 genome. Isothermal plasmid assembly [27] was prepared with these four DNA fragments and pJC4, which had been digested with *Xba*I and *Not*I before. The sequence integrity was verified by sequencing with primers ABseq 1–5.

Plasmid pEC-XT99A [51] served as basis for the GCV system overproduction plasmid and was digested with *Ecl*136II and *Xba*I. The gene cluster *gcvPTH* was amplified from the *C.* *jeikeium* K411 genome [84] with primer pair gcv_Cjk_start_EcoRV/gcv_Cjk_MluI_XbaI and the resulting PCR product was digested with *Eco*RV and *Xba*I and ligated into the cut pEC-XT99A. After verification by sequencing, this intermediate plasmid served as basis for the second cloning step. The gene cluster *lipAB* was amplified from the *C.* *jeikeium* K411 genome with primer pair lipB-Cjk_start-*Eco*RV/lipA-Cjk_stop-*Xba*I and the PCR product was digested with *Ssp*I und *Eco*RV, and ligated into the *Xmn*I cut intermediate plasmid to give pEC-XT99A_gcv_OP1-Cjk, which was verified by sequencing.

Plasmid pEC-XT99A_gcv_OP1-Cjk was transformed into strain *C.* *glutamicum* HIS8 resulting in *C.* *glutamicum* HIS9. The exchange of the translational start codon ATG of gene *pgi* to GTG [7] in *C.* *glutamicum* HIS9 was done with pK19*mobsacB* *pgi*^GTG^. For the construction, the flanking regions were amplified with primer pairs pgi1/pgi2 and pgi3/pgi4, containing the nucleotide exchange. The PCR products were used in a SOEing PCR with primer pair pgi1/pgi4. Then, the SOEing PCR and the vector were digested with *Hin*dIII and *Bam*HI and ligated together. After sequence verification, the plasmid was introduced into *C.* *glutamicum* HIS9; the base exchange was done as described above, and verified by sequencing with primer pgiseq.

### Intracellular adenylate measurements and energy charges

The determination of intracellular adenylate concentrations was done with a modified protocol as has been described before for *E.* *coli* [21]. 1 mL of culture suspension was collected during exponential growth phase (OD_600_ of 7.5) and sampled directly in − 20 °C cold 0.25 mL 35% (v v^−1^) perchloric acid containing 80 µM ethylenediaminetetraacetic acid (EDTA). The suspension was incubated for 15 min at 4 °C on a shaker, 0.25 mL 1 M K_2_HPO_4_ was added, and the suspension was neutralized with 5 M KOH to pH 7.0. After centrifugation (5 min, 4 °C, 7000×*g*), the supernatant was analyzed via HPLC (1200 series, Agilent Technologies, Santa Clara, CA, USA) equipped with a RP-C18 (octadecyl) phase column (Supelcosil LC-18-T, 3 µm, 150 cm × 4.6 mm) and a diode array detector (DAD). Buffer A (0.1 M KH_2_PO_4_, 0.1 M K_2_HPO_4_, 4 mM tetrabutylammonium bisulfate [TBAS], pH 6.0) and buffer B (0.1 M KH_2_PO_4_, 0.1 M K_2_HPO_4_, 4 mM TBAS, pH 7.2 with 30% (v v^−1^) methanol) were used to generate a gradient (3.5 min, 0% B; 20 min, 30% B, 22 min 35% B; 40 min, 60% B; 48 min, 100% B; 55 min, 100% B, 60 min 0% B) with a flow rate of 1 mL min^−1^. The energy charge of intracellular adenylates was calculated as has been defined before [4, 5]. The energy charge of the adenylate pool as a regulatory parameter. Interaction with feedback modifiers. Biochemistry [4, 5], with the difference that AMP was omitted from calculations, since AMP concentrations were below the detection limit of the applied analytical system. As such, current EC values are intrinsically higher by estimated 10% compared to the original definition:

$${\text{EC}} = \frac{{\left[ {\text{ATP}} \right] + \left[ {0.5 {\text{ADP}}} \right]}}{{\left[ {\text{ADP}} \right] + \left[ {\text{ATP}} \right]}}$$

### Quantification of substrate and product concentrations

Substrates and products were quantified by harvesting 1 mL of cell suspension via centrifugation (12,100×*g*, 5 min, RT) at given time points. The supernatants were used for further analysis. The glucose concentration was determined with a test kit from Roche (Roche Diagnostics, Mannheim, Germany). Quantification of amino acids was performed with an Agilent 1200 series apparatus (Agilent Technologies, Santa Clara, CA, USA) equipped with an Agilent Zorbax Eclipse Plus C_18_ column (250 × 4.6 mm, 5 µm) protected by an Agilent Zorbax Eclipse Plus C_18_ guard column (12.5 × 4.6 mm, 5 µm). Automatic precolumn derivatization with *ortho*-phthaldialdehyde was followed by fluorometric detection (excitation at 230 nm and emission at 450 nm). The elution buffer consisted of a polar phase (10 mM Na_2_HPO_4_, 10 mM Na_2_B_4_O_7_, 0.5 mM NaN_3_, pH 8.2) and a nonpolar phase (45% [v v^−1^] methanol, 45% [v v^−1^] acetonitrile). Protocol details were described earlier [16]. Analytes were quantified using 200 µM l-ornithine as the internal standard to correct variabilities in analytes and a seven-point calibration curve for each component as an external reference standard.

### Flux balance analysis

Metabolic fluxes of *C.* *glutamicum* ATCC 13032 were investigated by flux balance analysis (FBA), applying different objective functions and constraints [10]. All computations were carried out with MATLAB 2015b (The MathWorks, Natick, MA, USA) and the COBRA Toolbox v3.0 with glpk solvers [76], using the genome-scale metabolic model (GEM) of *C.* *glutamicum* ATCC 13032, iCW773 [98]. The glucose uptake rate was set to 3.94 mmol g_CDW_^−1^ h^−1^ for all simulations [12]; however, objective functions and constraints were changed as follows: (a) Maximizing growth rate with no further constraints results in *μ* = 0.36 h^−1^ and (b) maximizing l-histidine yield with a fixed *μ* of 0.1 h^−1^ resulted in a maximum yield of 0.51 mol l-histidine mol^−1^ glucose.

### Systems metabolic profiling (SMP)

#### Cultivation and extraction of metabolites

*Corynebacterium glutamicum* strains HIS1, HIS6, HIS7, HIS8, HIS9, and HIS10 were cultivated as described above. Sampling was performed at a CDW of approximately 1.8 g L^−1^ during the exponential growth phase. 2 mL of cell suspension was sampled by centrifugation (12,100×*g*, 20 s, 30 °C) and washed with 1.5 mL of 0.9% (w v^−1^) NaCl solution followed by centrifugation. Cells were quenched immediately with liquid nitrogen and temporarily stored at − 70 °C. Defined amounts of 250 μM l-norvaline solution (internal standard) were added to the cell pellets to obtain an extraction concentration of 20 g_CDW_ L^−1^. Immediately after addition, suspensions were pre-incubated for 30 s at 100 °C in a water bath and homogenized by vortexing (20 s). Subsequently, samples were incubated for 3 min at 100 °C, chilled on ice and centrifuged (20,800×*g*, 10 min, 4 °C). Supernatants were stored at − 70 °C [14].

#### LC-QTOF analysis of intracellular metabolites

Differential metabolite analysis was performed on an Agilent 1260 bio-inert HPLC coupled to an Agilent 6540 Accurate-Mass LC–MS/MS Q-TOF system with ESI Jet Stream Technology (Agilent Technologies, Santa Clara, CA, USA). Two different hydrophilic interaction chromatography (HILIC) systems were used to get high metabolite coverage. The first method was ammonium acetate based (10 mM, pH 9.2) utilizing a Sequant ZIC-pHILIC column (150 × 2.1 mm, 5 μm) with guard column (Sequant ZIC-pHILIC, 20 × 2.1 mm, 5 μm) at 40 °C, 0.2 mL min^−1^, and 5 μL injection volume. For details see [86]. Additionally, an acidic HILIC method was established using ammonium formate buffer (10 mM, pH 2.8) and a Waters XBridge BEH Amide column (150 × 2.1 mm, 3.5 μm) coupled to a Waters XBridge BEH Amide VanGuard Cartridge (5 × 2.1 mm, 3.5 μm) at 35 °C, 0.2 mL min^−1^, and 5 μL injection volume. Mobile phases were composed as follows: Mobile phase A: 90% acetonitrile/10% water, 10 mM ammonium formate and mobile phase B: 10% acetonitrile/90% water, 10 mM ammonium formate. Both adjusted to pH 2.8 with formic acid. Gradient elution was carried out by the following program: Isocratic hold 0% B for 1 min, linear gradient to 62.5% B for 15 min, linear gradient to 100% B for 4 min, column wash at 100% B for 5 min, linear gradient to 0% B for 5 min and column equilibration at 0% B for 15 min. Samples were prepared in 60% (v v^−1^) acetonitrile and 10 mM ammonium acetate (pH 9.2) or ammonium formate (pH 2.8). All metabolite samples were separated with both HILIC methods in positive and negative MS mode (tuned in extended dynamic range) with following conditions: drying gas flow rate of 8 L min^−1^ with a gas temperature of 325 °C, nebulizer with 40 lb per square inch gauge, sheath gas flow rate of 12 L min^−1^ and sheath gas temperature of 350 °C, capillary voltage of 4000 V and fragmentor voltage of 100 V. Additionally, fragmentation experiments in the targeted MS/MS mode were carried out to investigate and verify structure integrity of IGP, adenylosuccinate, SAICAR, and FGAR. For this, precursor ions [M+H] or [M−H], verified by accurate mass, were selected and fragmented at their characteristic retention times via collision-induced dissociation (CID) at 10, 20, and 30 V. Since analytical standards of those compounds were not commercially available or only by custom synthesis, fragmentation patterns were computationally evaluated with MassHunter Molecular Structure Correlator (B05.00, Agilent Technologies, Santa Clara, CA, USA). By combining accurate mass and plausible fragmentation patterns IGP, adenylosuccinate, SAICAR, and FGAR could be identified.

#### Data analysis

System control and acquisition were performed using MassHunter Data Acquisition (B06.01, Agilent Technologies, Santa Clara, CA, USA). As first step, an untargeted differential analysis was carried out to generate hypothesis free data. Peak picking and integration were done in MassHunter ProFinder (B08.00, Agilent Technologies, Santa Clara, CA, USA) using “batch recursive feature extraction”. Subsequently, statistical analysis was performed in Mass Profiler Professional (13.1.1, Agilent Technologies, Santa Clara, CA, USA). Significance testing was done by one-way ANOVA and *p* values were filtered (*p* < 0.05). Peaks were identified by accurate mass and with a personal compound data library, containing retention times of authentic standards. Unidentified significant features were searched against the METLIN [30] and MassBank [37] database. After identification, peak integration was manually curated via “batch targeted feature extraction”. The following metabolites of the de novo purine and l-histidine biosynthesis could be identified and analyzed with the applied method: fGAR, phosphoribosyl-*N*-formylglycineamide; SAICAR, phosphoribosyl-aminoimidazolesuccinocarboxamide; AICAR, 1-(5-phosphoribosyl)-5-amino-4-imidazolecarboxamide; IMP, inosine monophosphate; AdSucc, adenylosuccinate; ADP, adenosine diphosphate; ATP, adenosine triphosphate; IGP, imidazole-glycerol phosphate; l-histidinol and l-histidine.

## Appendix

See Table 2 and Fig. 7.

**Table 2:** Oligonucleotides used in this study

Oligonucleotide	Sequence	Purpose
hisG1	5′-CGCGGATCCCAGCTCGATTTGGGTATCACCG-3′	Outer primer #1 for introduction of *hisG*^G233H−T235Q^, *Bam*HI site underlined
hisG2	5′-CCAGTTGTCGCGTGCCAGTGGGGATACCTGTGGATGGGATAAGCCTGGGGTTACTGC-3′	Inner primer #1 for introduction of *hisG*^G233H−T235Q^, affected triplets underlined
hisG3	5′-GCAGTAACCCCAGGCTTATCCCATCCACAGGTATCCCCACTGGCACGCGACAACTGG-3′	Inner primer #2 for introduction of *hisG*^G233H−T235Q^, affected triplets underlined
hisG4	5′-CGCGGATCCGTTGATGGTGGTTCGTGAGATTTGG-3′	Outer primer #2 for introduction of *hisG*^G233H−T235Q^, *Bam*HI site underlined
hisGseq	5′-GGTATCCATCAAGCTTGG-3′	Sequencing primer for *hisG*^G233H−T235Q^
hisD1	5′-CCCAAGCTTCGGTGTCGCTGAAGTTAAGTTCTG-3′	Outer primer #1 for exchange of native promoter with P_*tuf*_ in operon *hisD*–*hisC*–*hisB*–*cg2302*–*cg2301*, *Hin*dIII site underlined
hisD2	5′-CGCAGGTCAGTGACATTCAACATATGTATTATGCATCTCGACCACCCCAGATTTACCTG-3′	Inner primer #1 for exchange of native promoter with P_*tuf*_ in operon *hisD*–*hisC*–*hisB*–*cg2302*–*cg2301*, *Nde*I and *Nsi*I sites underlined
hisD3	5′-CAGGTAAATCTGGGGTGGTCGAGATGCATAATACATATGTTGAATGTCACTGACCTGCG-3′	Inner primer #2 for exchange of native promoter with P_*tuf*_ in operon *hisD*–*hisC*–*hisB*–*cg2302*–*cg2301*, *Nde*I and *Nsi*I sites underlined
hisD4	5′-CGCGGATCCCTCATCAACACCCAAGATGGAAC-3′	Outer primer #2 for exchange of native promoter with P_*tuf*_ in operon *hisD*–*hisC*–*hisB*–*cg2302*–*cg2301*, *Bam*HI site underlined
tuf1	5′-AACTGCAGAACCAATGCATCCACAGGGTAGCTGGTAGTTTG-3′	Primer #1 for amplification of P_*tuf*_, *Nsi*I site underlined
tuf2	5′-GGGAATTCCATATGATGTCCTCCTGGACTTCGTGGTGGC-3′	Primer #2 for amplification of P_*tuf*_, *Nde*I site underlined
hisH1	5′-CCCAAGCTTCGGAGTGAAATGAGGTCCTTGGTC-3′	Outer primer #1 for exchange of native promoter with P_*tuf*_ in operon *hisH*–*hisA*–*impA*–*hisF*–*hisI*–*cg2294*, *Hin*dIII site underlined
hisH2	5′-GAGAAGGGCGACAGTTTTGGTCATATGTATTATGCATCTCCCTTCCTGAGAATGACGGCTAGTCG-3′	Inner primer #1 for exchange of native promoter with P_*tuf*_ in operon *hisH*–*hisA*–*impA*–*hisF*–*hisI*–*cg2294*, *Nde*I and *Nsi*I sites underlined
hisH3	5′-CGACTAGCCGTCATTCTCAGGAAGGGAGATGCATAATACATATGACCAAAACTGTCGCCCTTCTC-3′	Inner primer #1 for exchange of native promoter with P_*tuf*_ in operon *hisH*–*hisA*–*impA*–*hisF*–*hisI*–*cg2294*, *Nde*I and *Nsi*I sites underlined
hisH4	5′-CGCGGATCCGCACGAAGTAGAAACGCTCATCAGG-3′	Inner primer #2 for exchange of native promoter with P_*tuf*_ in operon *hisH*–*hisA*–*impA*–*hisF*–*hisI*–*cg2294*, *Bam*HI site underlined
hisN1	5′-CCCAAGCTTCGCATGAGCATTTCAGCCCAGTCC-3′	Outer primer #1 for exchange of native promoter with P_*tuf*_ in operon *cg0911*–*hisN*, *Hin*dIII site underlined
hisN2	5′-GCTCTGGATTAGTCATGCCTTCCATATGTATTATGCATCTCGGTGGCGTTCTAAGAATCACAATCG-3′	Inner primer #1 for exchange of native promoter with P_*tuf*_ in operon *cg0911*–*hisN*, *Nde*I and *Nsi*I sites underlined
hisN3	5′-CGATTGTGATTCTTAGAACGCCACCGAGATGCATAATACATATGGAAGGCATGACTAATCCAGAGC-3′	Inner primer #2 for exchange of native promoter with P_*tuf*_ in operon *cg0911*–*hisN*, *Nde*I and *Nsi*I sites underlined
hisN4	5′-CGCGGATCCCACCGAACCACGTATAACCCATGG-3′	Outer primer #2 for exchange of native promoter with P_*tuf*_ in operon *cg0911*–*hisN*, *Bam*HI site underlined
hisE1	5′-CCCAAGCTTCTGACAGAGTTGAAGGCCCTCGAG-3′	Outer primer #1 for exchange of native promoter with P_*dapA*–A16_ and change of the translational start codon from GTG to ATG in operon *hisE*–*hisG*, *Hin*dIII site underlined
hisE2	5′-CGTACAGCGAGTCAAATGTCTTCA**T**ATGTATTATGCATCTCGGAGGATATTAGTCGAATAATTTC-3′	Inner primer #1 for exchange of native promoter with P_*dapA*–A16_ and change of the translational start codon from GTG to ATG in operon *hisE*–*hisG*, *Nde*I and *Nsi*I sites underlined, base exchange bold
hisE3	5′-GAAATTATTCGACTAATATCCTCCGAGATGCATAATACAT**A**TGAAGACATTTGACTCGCTGTACG-3′	Inner primer #2 for exchange of native promoter with P_*dapA*–A16_ and change of the translational start codon from GTG to ATG in operon *hisE*–*hisG*, *Nde*I and *Nsi*I sites underlined, base exchange bold
hisE4	5′-CGCGGATCCGCAACGTAGATGGCGATATCTTTAGG-3′	Outer primer #1 for exchange of native promoter with P_*dapA*–A16_ and change of the translational start codon from GTG to ATG in operon *hisE*–*hisG*, *Bam*HI site underlined
dapA1	5′-AACTGCAGAACCAATGCATTGGTTCTGCAGTTATCACACCC-3′	Primer #1 for amplification of P_*dapA*–A16_, *Nsi*I site underlined
dapA2	5′-GGGAATTCCATATGAGGCTCCTTTTAAATCGAGCGGCTCCGGTCTTAGCTGTTAAACC-3′	Primer #2 for amplification of P_*dapA*–A16_, *Nde*I site underlined
hisF1	5′-CGCAAGCTTCTCCATGCCCATGCTGGGTAAACGC-3′	Primer #1 for integration of stop codon and P_*sodA*_ in front of *hisF*, *Hin*dIII site underlined
hisF2	5′-GTCACAAGCCCGGAATAATTGGCAG**TTA**TTTACTTGTACTCCTCATTTAACG-3′	Primer #2 for integration of stop codon and P_*sodA*_ in front of *hisF*, stop codon bold
sodA1	5′-CGTTAAATGAGGAGTACAAGTAAA**TAA**CTGCCAATTATTCCGGGCTTGTGAC-3′	Primer #3 for integration of stop codon and P_*sodA*_ in front of *hisF*, stop codon bold
sodA2	5′-GAATAACTCGAATTGCCACGCCCATGGGTAAAAAATCCTTTCGTAGGTTTC-3′	Primer #4 for integration of stop codon and P_*sodA*_ in front of *hisF*
hisF3	5′-GAAACCTACGAAAGGATTTTTTACCCATGGGCGTGGCAATTCGAGTTATTC-3′	Primer #5 for integration of stop codon and P_*sodA*_ in front of *hisF*
hisF4	5′-CGCGGATCCCGCCGCGCTTTGCCCACTCGATTGC-3′	Primer #6 for integration of stop codon and P_*sodA*_ in front of *hisF*, *Bam*HI site underlined
hisFseq	5′-CTGACACCGAAGGCCATC-3′	
tuf2_1	5′-GATCAGCGACGCCGCAGGGTCTAGACCACAGGGTAGCTGGTAGTTTGAAAATC-3′	Primer #1 for amplification of P_*tuf*_, *Xba*I site underlined
tuf2_2	5′-CATTGAGCGCCGACAATAACGATTGCAGCCATTGTATGTCCTCCTGGACTTCG-3′	Primer #2 for amplification of P_*tuf*_
purA1	5′-CGAAGTCCAGGAGGACATACAATGGCTGCAATCGTTATTGTCGGCGCTCAATG-3′	Primer #1 for amplification of *purA* (cg3063)
purA2	5′-GATCTTCTTTTTATCAGCCACTGTATGTCCTCCTGGACTTCCTAGTTGTCAGCTAGTACG-3′	Primer #2 for amplification of *purA* (cg3063)
purB1	5′-CGTACTAGCTGACAACTAGGAAGTCCAGGAGGACATACAGTGGCTGATAAAAAGAAGATC-3′	Primer #1 for amplification of *purB* (cg2876)
purB2	5′-CTTCTCTCATCCGCCAAAACAGTTAAAGAATCTCACCTGGTCGGTAGTC-3′	Primer #2 for amplification of *purB* (cg2876)
rrnB1	5′-GACTACCGACCAGGTGAGATTCTTTAACTGTTTTGGCGGATGAGAGAAG-3′	Primer #1 for amplification of terminator region of *rrnB* (*E. coli*)
rrnB2	5′-CTGCAGATATCCATCACACTGGCGGCCGCAGGAGAGCGTTCACCGACAAACAAC-3′	Primer #2 for amplification of terminator region of *rrnB* (*E. coli*), *Not*I site underlined
ABseq1	5′-GCGATTGAAGACCGTC-3′	Sequencing primer for pJC4 *purA purB*
ABseq2	5′-GCAATCGGCACCACCGGC-3′	Sequencing primer for pJC4 *purA purB*
ABseq3	5′-GCCTGCATGGGACGAAG-3′	Sequencing primer for pJC4 *purA purB*
ABseq4	5′-GTCACCGAACTGCTCAAC-3′	Sequencing primer for pJC4 *purA purB*
ABseq5	5′-GATGGAAGCAGGATCGCG-3′	Sequencing primer for pJC4 *purA purB*
pgi1	5′-CCCAAGCTTCAGCGTTGCGTGACGCACTCATTG-3′	Outer primer #1 for introduction of *pgi*^GTG^, *Hin*dIII site underlined
pgi2	5′-CCAAACCTGGGTGGTCGAAATGTCCGCCA**C**GAAAACTCCTTTATTGTCG-3′	Inner primer #1 for introduction of *pgi*^GTG^, nucleotide exchange bold
pgi3	5′-CGACAATAAAGGAGTTTTC**G**TGGCGGACATTTCGACCACCCAGGTTTGG-3′	Inner primer #2 for introduction of *pgi*^GTG^, nucleotide exchange bold
pgi4	5′-CGCGGATCCCCAATGTTGACGATCTTCTTGATCG-3′	Outer primer #2 for introduction of *pgi*^GTG^, *Bam*HI site underlined
pgiseq	5′-CAAGCGTTGGGTTAAGGAGGA-3′	Sequencing primer for *pgi*^GTG^
gcv_Cjk_start_*Eco*RV	5′-ATCGATATCCGAGAGGAGACACAACATGTCTTCTGCAGCTACTCGC-3′	Primer #1 for amplification of gene cluster *gcvPTH* from *C.* *jeikeium* genome
gcv_Cjk_*Mlu*I_XbaI	5′-CAGTCTAGAACGCGTGGAACCGACCATAGGGTCTTG-3′	Primer #2 for amplification of gene cluster *gcvPTH* from *C.* *jeikeium* genome
lipB-Cjk_start-*Eco*RV	5′-GCGGATATCATGGGATTCCAGCAAGGC-3′	Primer #1 for amplification of gene cluster *lipAB* from *C.* *jeikeium* genome
lipA-Cjk_stop-*Xba*I	5′-GCGTCTAGATCCTTCGCCATGGATTCAAC-3′	Primer #2 for amplification of gene cluster *lipAB* from *C.* *jeikeium* genome
pK19-fw	5′-TAATGCAGCTGGCACGAC-3′	Primer #1 for sequencing the pK19*mobsacB* inserts
pK19-rev	5′-GTAGCTGACATTCATCCG-3′	Primer #2 for sequencing the pK19*mobsacB* inserts
